# The antimicrobial potential of the secretome from Wharton’s jelly mesenchymal stem cells in the context of regenerative medicine: an *in vitro* study

**DOI:** 10.3389/fimmu.2026.1818127

**Published:** 2026-05-05

**Authors:** Filomena Napolitano, Emanuela Roscetto, Maria Fabozzi, Martina Aversa, Pietro Formisano, Maria Rosaria Catania, Nunzia Montuori

**Affiliations:** 1 Department of Translational Medical Sciences, University of Naples Federico II, Naples, Italy; 2 Department of Molecular Medicine and Medical Biotechnology, University of Naples Federico II, Naples, Italy; 3 Center for Basic and Clinical Immunology Research (CISI), WAO Center of Excellence, University of Naples Federico II, Naples, Italy

**Keywords:** antimicrobial peptides, bacterial infection, immunomodulation, stem cell secretome, tissue regeneration, Wharton’s jelly

## Abstract

**Introduction:**

The secretome derived from Mesenchymal Stem Cells (MSCs) have received significant attention as a promising tool in regenerative medicine. This “cell-free” strategy offers a promising alternative to stem cell therapies, potentially avoiding issues like immunogenicity and tumorigenicity. Recently, we demonstrated that MSC-conditioned media (MSC-CM) obtained from MSCs isolated from Wharton’s jelly (WJ) exerts notable bioactive effects associated with wound healing and tissue repair. Growing evidence suggests that WJ-MSC-CM have potential antiseptic effects through their ability to produce antimicrobial factors and modulate immune responses. In this context, further characterization of WJ-MSC-CM is essential to clarify its role in modulating oxidative stress and host defense responses, thereby supporting its potential application in regenerative therapies.

**Methods:**

We analyzed the effects of WJ-MSC-CM on reactive oxygen species (ROS) generation and antioxidant system activity in dermal fibroblasts subjected to hydrogen peroxide (H_2_O_2_) exposure. Cellular metabolism assays were used to analyze antioxidant responses. In addition, we assessed the antibacterial potential against different bacterial strains and assessed the content of antimicrobial peptides in WJ-MSC-CM by ELISA.

**Results:**

WJ-MSC-CM-treated fibroblasts exhibited elevated ROS levels following H_2_O_2_ exposure yet displayed an upregulation of antioxidant systems in fibroblast homogenate samples. Moreover, WJ-MSC-CM protected against H_2_O_2_-induced cytotoxicity. WJ-MSC-CM contained significant levels of antimicrobial peptides and demonstrated inhibitory activity against a Gram-positive bacterium such as *Staphylococcus aureus*.

**Conclusion:**

These findings emphasize the antiseptic potential of WJ-MSC-CM and support its relevance as a promising cell-free strategy in regenerative medicine.

## 1 Introduction

The umbilical cord stroma contains a distinctive gelatinous connective tissue known as Wharton’s jelly (WJ), which is notable for its abundance of mesenchymal stem cells (MSCs).

WJ-MSCs have gained increasing attention in recent years due to several advantages over MSCs derived from other sources. These include faster proliferation rates, greater *ex vivo* expansion capacity, a lower risk of graft-versus-host disease, and no associated risk of teratoma formation (1–4). To date, hundreds of clinical trials have been registered on ClinicalTrials.gov investigating the use of WJ-MSCs for the treatment of a wide range of diseases, showing encouraging therapeutic potential.

As increasing attention shifts from cell transplantation toward secretome-based approaches as alternative therapeutic strategies, numerous studies have demonstrated that WJ-MSCs exhibit a uniquely distinctive secretome compared with MSCs derived from other tissue sources, particularly in key functional domains such as anti-inflammatory activity and angiogenesis (5). The bioactive molecules and factors present in the secretome encompass a wide range of components, including cytokines, chemokines, growth factors, angiogenic mediators, hormones, integrins, metabolites, and regulatory nucleic acids (6). Although MSCs from different tissues share common characteristics including the expression of common cell surface markers (CD105, CD73 and CD90) and multipotency capacity to differentiate into osteoblasts, chondrocytes, or adipocytes, they have different secretive properties and the unique properties of WJ-MSCs have drawn considerable attention as an alternative stem cell source for regenerative medicine (5, 7).

A recent comparative secretome profiling by ELISA revealed that WJ-MSCs secrete higher levels of FGF-2, VEGF, IL-6, and SDF, and display greater potency than bone marrow-derived human mesenchymal stromal cells (BM-MSCs) in regulating inflammation, immunomodulation, angiogenesis, and fibrosis (8). In the study by Napolitano et al, it was found that WJ-MSCs actively produced and released through the secretome various cytokines, chemokines, and growth factors more efficiently than MSCs isolated from other tissues, including bone marrow, adipose tissue, and cord blood (9). Furthermore, the unique composition of the WJ-MSC secretome promotes dermal fibroblast proliferation, migration, and wound repair, and provides protection against cellular senescence. In an *in vivo* rat acute wound model, WJ-MSC secretome applied to the back of test animals significantly promoted re-epithelialization, vascularization and granulation maturation (10). Ongoing research is increasingly directed toward the development of gel carriers capable of delivering the WJ-MSC secretome, with the aim of enhancing cellular regeneration and accelerating wound healing processes. Preliminary studies demonstrated the efficacy of this approach, suggesting its potential as a therapeutic option for diabetic foot ulcers (11).

Over the past few years, numerous studies have highlighted that MSCs possess significant antimicrobial properties in addition to their well-established regenerative functions. These properties arise from both direct and indirect mechanisms. Directly, MSCs can secrete a variety of antimicrobial peptides and factors, including cathelicidins, defensins, and lipocalin-2, which can inhibit the growth of bacteria and fungi. Indirectly, MSCs modulate the immune microenvironment, enhancing the activity of innate immune cells such as neutrophils and macrophages, thereby strengthening host defense responses (12–14). Recent research has particularly focused on the antibacterial potential of the MSC secretome, showing that cell-free strategy can exert potent antimicrobial effects. Adipose tissue-derived MSCs (AT-MSC) secretome exerted antibacterial effects against *Staphylococcus* spp. clinical strains (12). Non-preconditioned BM-MSC secretome inhibited *Vibrio cholerae* growth and biofilm formation *in vitro* (15). However, none of these preliminary studies have examined the antimicrobial activity of WJ-MSC secretome. Therefore, this study aims to explore the antibacterial potential of WJ-MSC-CM on different strains. This will be first achieved through an examination of the pro- and antioxidant properties, as these functions play a critical role in modulating cellular stress responses and may contribute to the elimination of pathogens. By characterizing the redox-modulating profile of WJ-MSC secretome, we aim to determine how these properties influence overall antibacterial activity, as well as to identify and analyze the key antimicrobial peptides responsible for these effects.

## 2 Methods

### 2.1 Cell culture

WJ-MSCs, BM-MSCs, and cord blood (CB)-MSCs were isolated from different donors and characterized for all standard MSC properties, as previously described (9). Briefly, cells were cultured in α-MEM supplemented with 1% Glutagro™ (Corning) and 10% FCS (Gibco) or cryopreserved in medium supplemented with 10% FCS (Gibco) containing 10% DMSO (Sigma-Aldrich) at −80 °C until use. Cells were routinely screened for Mycoplasma contamination. BJ (human foreskin fibroblasts; ATCC CRL-2522) were cultured in DMEM (Lonza) with 10% FCS (Gibco), 1% L-glutamine (Corning), at 37 °C in a humidified atmosphere containing 5% CO_2_.

### 2.2 Conditioned medium and lysate collection

The collection of conditioned medium was performed as previously described (9). Briefly, MSCs were seeded at 5 × 10^5^ cells per P60 plate and incubated at 37 °C with 5% CO_2_ for 24 h to allow cell adhesion. The growth medium was then discarded, and the cells were washed three times with PBS to remove residual serum proteins. Subsequently, the cells were cultured in basal medium without serum. After 24 h of incubation, the supernatants were collected and centrifuged at 5000 rpm for 10 minutes to remove cellular debris. The conditioned media were stored at −80 °C until use.

For lysate collection, WJ-MSCs were washed twice with PBS and harvested by scraping in cold lysis buffer (50 mM HEPES, 150 mM NaCl, 10% glycerol, 1% Triton X-100, 1 mM EGTA, 1.5 mM MgCl_2_, 10 mM NaF, 10 mM sodium pyrophosphate, and 1 mM Na_3_VO_4_) supplemented with a cocktail of proteases and phosphatases inhibitors. Cell suspensions were incubated on ice for 30 min with occasional gentle mixing and then centrifuged at 12,000 rpm for 15 min at 4 °C to remove insoluble material. The supernatants, representing the cell lysates, were collected and stored at −20 °C until further analysis.

### 2.3 ROS production

BJ fibroblasts were seeded at 2 × 10^4^ cells/well in 96-well plates and allowed to adhere overnight in DMEM supplemented with 10% FBS. Intracellular ROS levels were assessed using 2′,7′-dichlorodihydrofluorescein diacetate (DCFH-DA). Cells were incubated with 5 μM DCFH-DA for 30 minutes at 37 °C in the dark. DCFH-DA, a cell-permeable nonfluorescent probe, is deacetylated by intracellular esterases to form dichlorodihydrofluorescein (DCFH), which is subsequently oxidized by ROS to yield the fluorescent product dichlorofluorescein (DCF). Following incubation, cells were washed twice and then treated with either control medium or WJ-MSC-CM, in the presence or absence of 1 mM H_2_O_2_, for 5, 15, 30, or 60 minutes at 37 °C in a humidified 5% CO_2_ incubator. Fluorescence intensity (DCF) was measured using a microplate reader (Tecan Trading AG, Switzerland) at an emission wavelength of 535 nm.

### 2.4 Cell viability assay

BJ fibroblasts were serum-starved overnight in DMEM supplemented with 0.1% BSA, plated at 5×10^3^ cells/well in 96-well plates, and then treated with WJ-MSC-CM containing H_2_O_2_ at different concentrations (1, 5, and 10 mM). Cells cultured in WJ-MSC-CM without H_2_O_2_ served as negative controls. Cell proliferation was assessed at 2, 4, 8, and 24 hours. At each point, 20 μL/well of the CellTiter 96^®^ AQueous One Solution Cell Proliferation Assay (Promega, Madison, WI, USA), a colorimetric assay that measures metabolically active viable cells, was added. After a 2-hour incubation at 37 °C, absorbance was measured at 490 nm using a microplate reader, following the manufacturer’s instructions.

### 2.5 Assessment of antioxidant enzyme activities

For antioxidant evaluation, cultured cells were collected and processed under cold conditions. A total of 1×10^6^ cells were harvested and homogenized in PBS using an ultrasonic cell disruptor. Homogenates were centrifuged at 10,000 × g for 10 minutes at 4 °C, and the resulting supernatants were used for biochemical assays. The enzymatic activities of catalase (CAT, Elabscience, Cat# E-BC-K031-M), glutathione peroxidase (GSH-Px, Elabscience, Cat# E-BC-K096-M), and total superoxide dismutase (T-SOD, Elabscience, Cat# E-BC-K020-M) were quantified using commercially available assay kits according to the manufacturers’ protocols. All enzyme activities were normalized to total protein concentration, which was determined using the BCA assay.

### 2.6 Bacterial strains and culture conditions

The bacterial strains used for the screening were methicillin-resistant *Staphylococcus aureus* (MRSA) ATCC 43300 and *Pseudomonas aeruginosa* ATCC 9027, obtained from American type Culture Collection (ATCC; Rockville, MD, USA). Bacteria were cultured on Trypticase Soy Agar II supplemented with 5% sheep blood (TSA; Becton Dickinson, USA) and incubated at 37 °C for 24 h under standard aerobic conditions. Species identification and antibiotic susceptibility profiles were determined using the BD Phoenix automated system (Becton Dickinson, Franklin Lakes, NJ, USA) and interpreted according to the guidelines of the European Committee on Antimicrobial Susceptibility Testing (EUCAST) (16).

### 2.7 Cell culture supernatants collection

BJ fibroblasts were seeded in 6-well plates at a density of 5 × 10^5^ cells per well in antibiotic-free growth medium and incubated for 24 h. Cells were then rinsed three times with 1× PBS and incubated with either growth medium alone or WJ-MSC-CM for 8 h, in the presence or absence of H_2_O_2_. Subsequently, cell culture supernatants were collected and centrifuged at 5000 rpm for 10 minutes to remove cellular debris. The conditioned media were stored at −80 °C until microbiological testing. The experimental design consisted of six experimental groups, as summarized in **Table 1**.

**Table 1:** Experimental scheme.

Group 1	Group 2	Group 3	Group 4	Group 5	Group 6
Growth medium (α-MEM) alone without antibiotic	WJ-MSC-CM alone	Cell culture supernatants from BJ fibroblasts cultured in growth medium without antibiotics	Cell culture supernatants from BJ fibroblasts cultured in growth medium without antibiotics and stimulated with H_2_O_2_ (1 mM)	Cell culture supernatants from BJ fibroblasts cultured in WJ-MSC-CM	Cell culture supernatants from BJ fibroblasts cultured in WJ-MSC-CM and stimulated with H_2_O_2_ (1 mM)

Analysis of the antibacterial activity of cell-conditioned media. For details see the “methods”.

### 2.8 Agar well-diffusion test

The antibacterial activity of cell culture supernatants against *S. aureus* and *P. aeruginosa* was performed through the agar well-diffusion method (17). Bacterial inocula were prepared in sterile physiological saline solution from overnight cultures of each test strain and adjusted to 0.5 McFarland standard of turbidity (approximately 1-2 × 10^8^ CFU/mL). Each suspension was uniformly spread onto Mueller–Hinton agar plates using sterile swabs. Wells of 8mm diameter were punched into the agar medium and filled with 50 μl of test cell culture supernatants. Vancomycin-impregnated disk (5 μg) and Meropenem-impregnated disk (10 μg) (D.I.D S.p.A., Italy) were placed on the agar surface as positive control for *S. aureus* and *P. aeruginosa*, respectively. Plates were incubated at 37 °C for 24 h under aerobic conditions. After incubation, the diameters of the inhibition zones surrounding the wells or antibiotic disks were measured in millimeters. All experiments were performed in triplicate.

### 2.9 Determination of the minimum inhibitory concentration

The antibacterial activities of cell culture supernatants against *S. aureus* were evaluated through standard broth microdilution method to determine the Minimum Inhibitory Concentration (MIC), defined as the lowest supernatant concentration at which no visible bacterial growth was detected. The bacterial inoculum was prepared in 2x Mueller Hinton Broth (MHB) and adjusted to a turbidity equivalent to 0.5 McFarland standard. The suspension was then diluted 1:100 and 100 μL of the resulting inoculum was dispensed into each well of a sterile 96-well microtiter plate. The test surnatantes were prepared in two-fold serial dilutions in sterile physiological saline solution and a volume of 100 μL of each dilution was added in triplicate to the wells containing the bacterial inoculum. Wells without surnatant were used as negative controls. Vancomycin (ranged from 0.25 µg/mL to 2 µg/mL) was included as the control. The plates were incubated under shaking at 37 °C overnight under aerobic conditions. *S. aureus* growth was quantified by measuring optical density at 590 nm using a microplate reader.

### 2.10 ELISA assays

The levels of antimicrobial peptides (AMPs) in WJ-MSC-CM were quantified using ELISA assay kits according to the manufacturers’ instructions. Culture medium alone was used as a negative control. Specifically, LL-37 (ELK Biotechnology, Cat# E-EL-ELK8189), Beta-Defensin-1 (Elabscience, Cat# E-EL-H0995), Beta-Defensin-2 (Elabscience, Cat# E-EL-H0996), and Hepcidin (Elabscience, Cat# E-EL-H6202) were quantified. Optical density was read at 450 nm using a BGMR-1000 microplate reader (Hangzhou Bio-Gener Technology Co., China), and peptide concentrations were calculated from standard curves generated with the standards provided in each kit.

### 2.11 Statistical analysis

Statistical analyses were performed using GraphPad Prism 9 for Windows (GraphPad Software, La Jolla, CA, USA). Data are presented as mean ± standard deviation (SD). Comparisons between groups were performed using an unpaired t-test or the Mann–Whitney U test, as appropriate. A *p* value < 0.05 was considered statistically significant.

## 3 Results

### 3.1 Differential regulation of ROS generation by WJ-MSC-CM under basal and oxidative stress conditions

**Figure 1:**
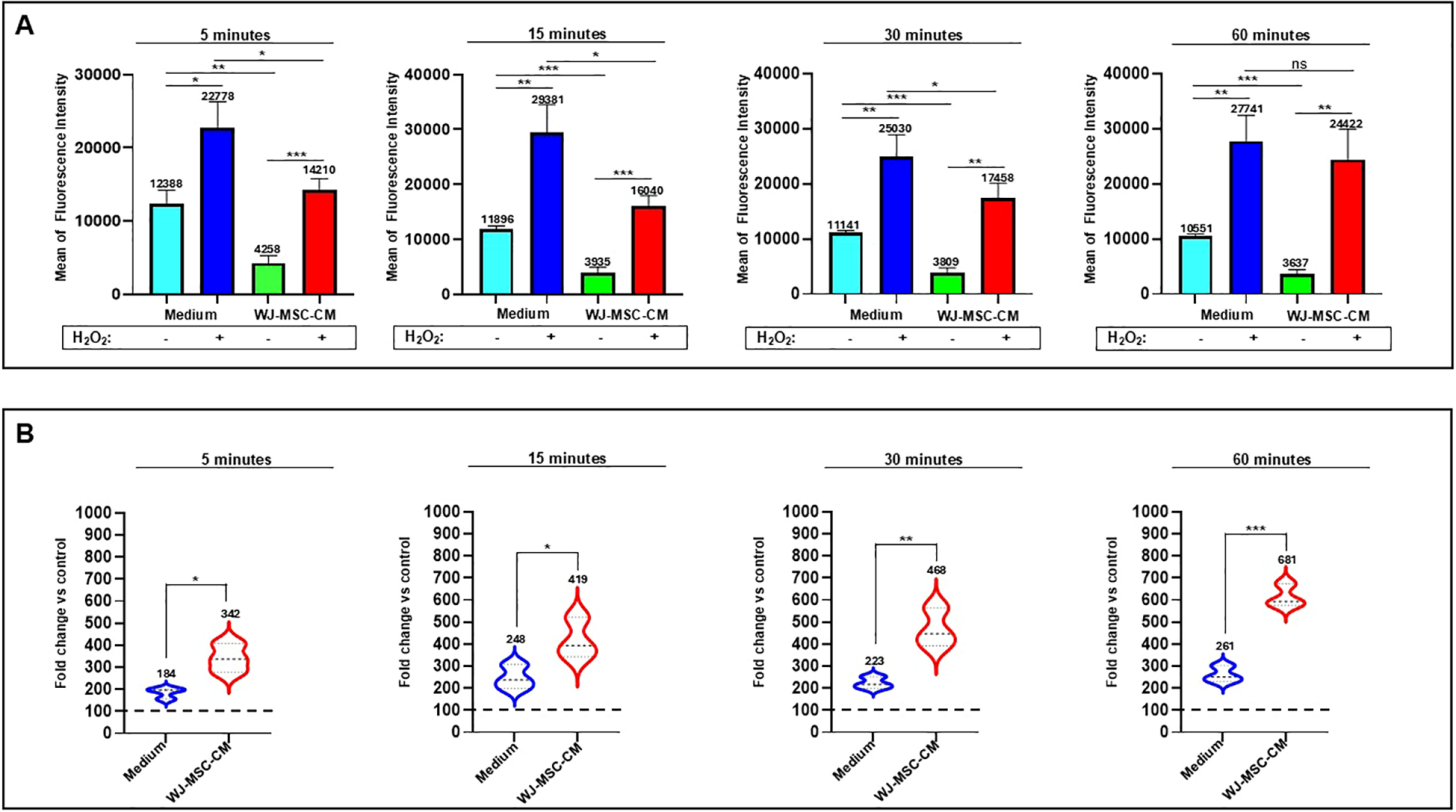
Effects of WJ-MSC-CM on intracellular ROS levels in dermal fibroblasts under oxidative stress. **(A)** Mean fluorescence intensity of intracellular ROS measured at 5, 15, 30, and 60 minutes in dermal fibroblasts cultured in basal medium or WJ-MSC-CM, in the absence (–) or presence (+) of H_2_O_2_. Data are expressed as mean ± SD. Statistical significance is indicated as *p < 0.05, **p < 0.01, and ***p < 0.001. **(B)** Violin plots showing fold change of ROS levels relative to untreated control cells at the indicated time points in fibroblasts cultured in basal medium or WJ-MSC-CM following H_2_O_2_ exposure. The dashed line represents the control baseline. Statistical significance is indicated as *p < 0.05, **p < 0.01, and ***p < 0.001.

We collected the conditioned medium from WJ-MSCs (WJ-MSC-CM) as a model of the secretome, containing both soluble factors and vesicular components, and investigated its effects on intracellular ROS levels in dermal fibroblasts. To this end, cells were stained with the DCFH-DA probe and incubated in the presence or absence of H_2_O_2_ as an exogenous oxidative stimulus. As shown in **Figure 1A**, mean fluorescence intensity was measured at 5, 15, 30, and 60 minutes under control conditions (medium) or following exposure to WJ-MSC-CM, with or without H_2_O_2_. At all-time points, cells cultured in medium alone exhibited markedly higher basal fluorescence levels compared with cells treated with WJ-MSC-CM. As expected, H_2_O_2_ treatment significantly increased fluorescence intensity in both medium- and WJ-MSC-CM-cultured cells. Notably, WJ-MSC-CM significantly attenuated the H_2_O_2_-induced increase in fluorescence at all time points, indicating a sustained protective effect against oxidative stress.

**Figure 1B** presents violin plots showing the fold change in fluorescence intensity of H_2_O_2_-treated cells relative to untreated controls for each condition. In medium-cultured cells, H_2_O_2_ induced a moderate, time-dependent increase in fluorescence. In contrast, cells exposed to WJ-MSC-CM displayed a significantly increase in fold change of fluorescence intensity at all examined time points (5, 15, 30, and 60 minutes). This suggests that WJ-MSC-CM acts as a modulatory agent, reducing basal intracellular ROS levels while allowing cells to exhibit a more efficient, time-dependent increase in ROS when exposed to exogenous stressors like H_2_O_2_.

Overall, these data indicate that WJ-MSC-CM could modulate oxidative stress by reducing harmful basal levels while enabling protective, transient, and time-dependent ROS spikes, as happens during bacterial infections.

### 3.2 WJ-MSC-CM protects fibroblasts from H_2_O_2_-induced cytotoxicity

**Figure 2:**
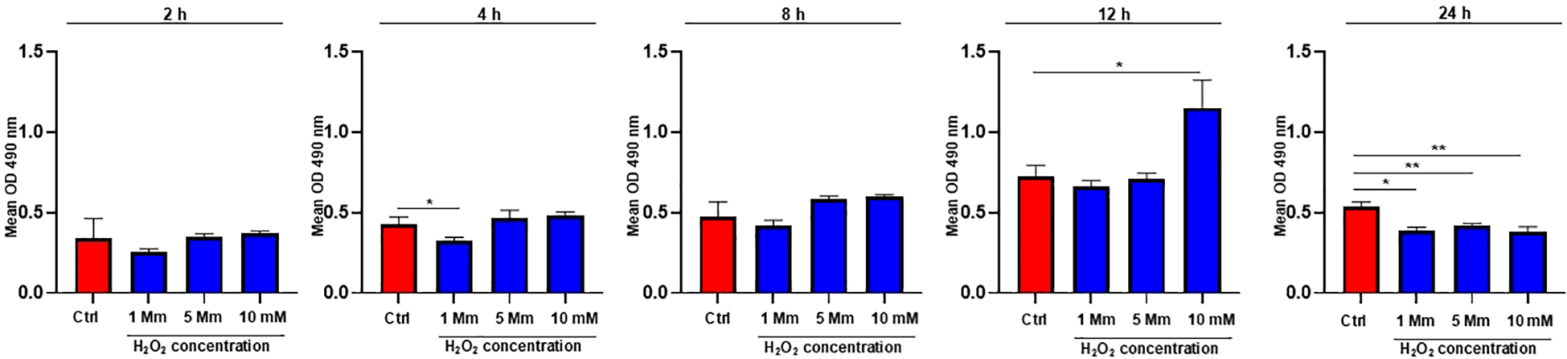
Effects of WJ-MSC-CM on H2O2-induced cytotoxicity in dermal fibroblasts. Bar graphs showing the mean optical density (OD) at 490 nm, measured by a colorimetric proliferation assay, in dermal fibroblasts exposed to increasing concentrations of H_2_O_2_ (1 mM, 5 mM, 10 mM) with WJ-MSC-CM treatment, compared to untreated control (Ctrl), at different time points (2, 4, 8, 12, and 24 hours). Data are expressed as mean ± SD. Statistical significance is indicated as *p < 0.05 and **p < 0.01.

On this basis, we evaluated cytotoxicity in H_2_O_2_-stressed cells cultured in WJ-MSC-CM to determine whether the observed modulation of ROS levels was associated with changes in cell viability. To this end, BJ cells were exposed to increasing concentrations of H_2_O_2_ (1, 5, and 10 mM), and cell viability was tested at 2, 4, 8, 12, and 24 hours using the MTS assay (**Figure 2**). At early time points (2 and 4 hours), no cytotoxic effects were observed, although a slight but significant reduction in cell viability was detected in cells exposed to 1 mM H_2_O_2_ at 4 hours (*p* = 0.02). At 8 hours, cell viability in H_2_O_2_-exposed cell groups remained comparable to control levels, indicating a transient cellular adaptation to oxidative stress. At the time of 12 hours, cells exposed to 10 mM H_2_O_2_ showed significantly higher optical density (OD) values compared to control (*p* = 0.01), suggesting a significant adaptive metabolic or growth response. At the time of 24 hours, cell viability in H_2_O_2_-exposed cell groups significantly decreased compared with control cells, both at 1 mM (*p* = 0.001), 5 mM (*p* = 0.002), and 10 mM (*p* = 0.003).

### 3.3 WJ-MSC-CM significantly boosts the activity of antioxidant enzymes

**Figure 3:**
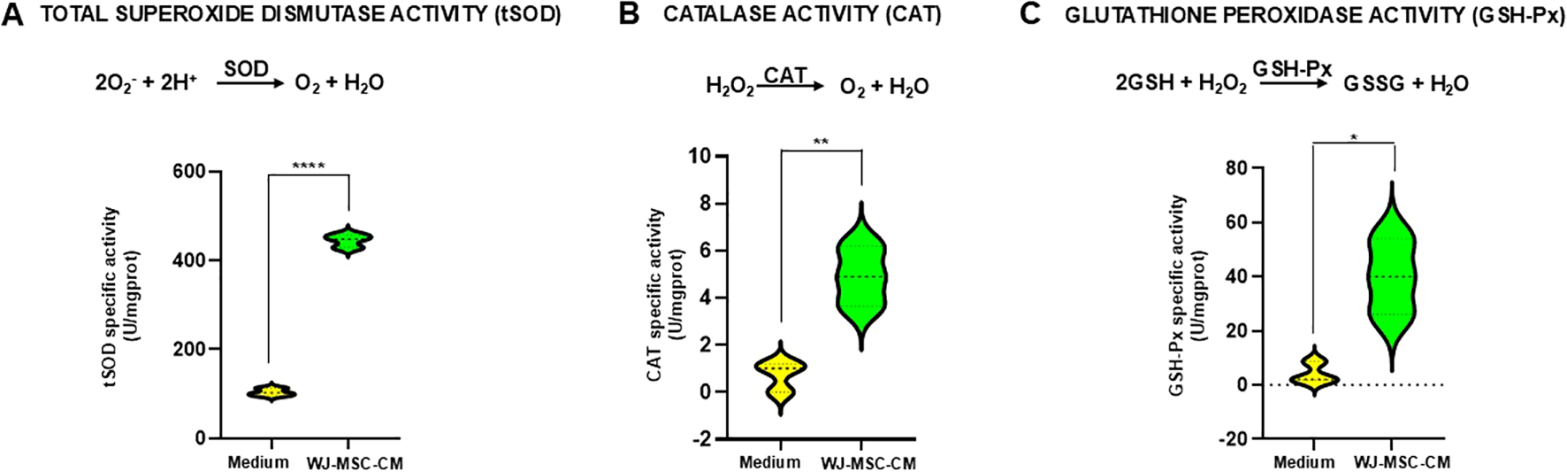
Effects of WJ-MSC-CM treatment on antioxidant enzyme activities in dermal fibroblasts. Enzymatic activity of **(A)** total superoxide dismutase (tSOD), **(B)** catalase activity (CAT), and **(C)** glutathione peroxidase activity (GSH-Px) in cell homogenates of dermal fibroblasts cultured in basal medium or treated with WJ-MSC-CM. Enzyme activities are expressed as international units per milligram of protein (U/mg). Statistical significance compared with the untreated control group is indicated as *p < 0.05, **p < 0.01, and ***p < 0.001.

Given that previous experimental evidence demonstrated that WJ-MSC-CM maintains low intracellular levels of ROS and protects dermal fibroblasts from H_2_O_2_-induced cytotoxicity, we next evaluated the effects of WJ-MSC-CM on the enzymatic activities of total SOD (tSOD), CAT, and GPx. Enzyme activities were assessed in dermal fibroblast homogenates following an 8-hour exposure to WJ-MSC-CM. As shown in **Figure 3A**, tSOD activity was markedly upregulated following WJ-MSC-CM exposure (445.1 U/mgprot), showing a highly significant increase (*p* = 0.0001) compared with controls (103.7 U/mgprot). Similarly, CAT activity was significantly increased (*p* = 0.0069) in fibroblast homogenates following WJ-MSC-CM treatment (4.913 U/mgprot) compared with control medium (0.0069 U/mgprot), as shown in **Figure 3B**. Notably, a significant increase (*p* = 0.013) in GPx activity was observed in WJ-MSC-CM–treated cells (40 U/mgprot) compared with control medium (4.2 U/mgprot), as illustrated in **Figure 3C**.

Overall, these findings indicate that WJ-MSC-CM plays a key role in regulating cellular redox homeostasis and enhancing stress resistance in dermal fibroblasts by promoting the activity of SOD, CAT, and GPx. This effect may contribute to the attenuation of ROS-induced DNA damage and inflammatory responses.

### 3.4 WJ-MSC-CM inhibits *Staphylococcus aureus* bacterial growth

**Figure 4:**
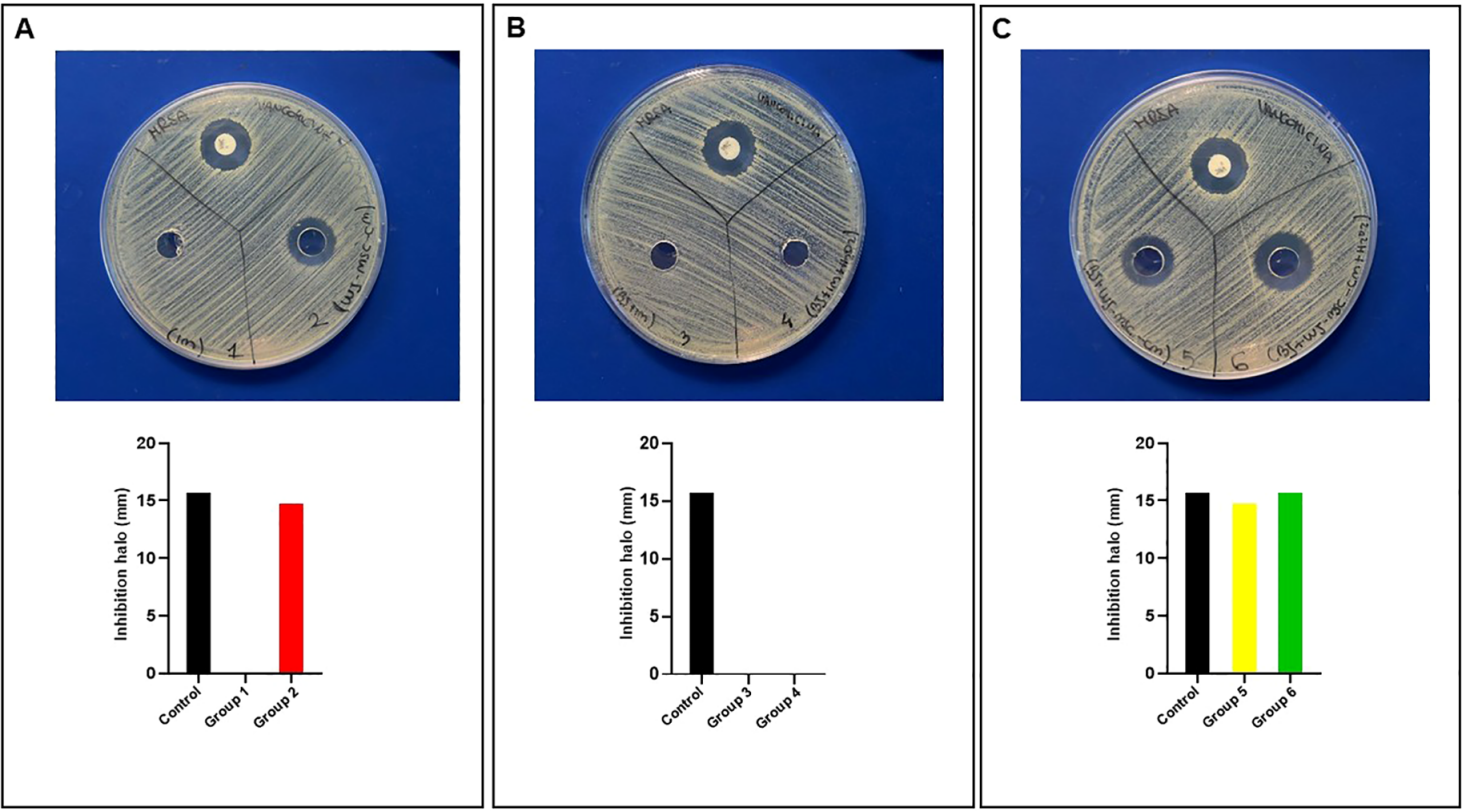
Analysis of antibacterial activity of WJ-MSC-CM against *S. aureus*. Representative images of agar diffusion assay plates inoculated with bacteria are shown for the experimental groups, including vancomycin as a positive control. Antibacterial activity was evaluated by the presence of a halo of inhibition, and the corresponding graph reports the diameter of the inhibition halo expressed in millimeters (mm): **(A)** culture medium alone (Group 1) and WJ-MSC-CM (Group 2); **(B)** conditioned media from untreated fibroblasts in the absence (Group 3) or presence (Group 4) of H_2_O_2_; and **(C)** conditioned media from WJ-MSC-CM–treated fibroblasts in the absence (Group 5) or presence (Group 6) of H_2_O_2_. Corresponding graphs report the diameter of the inhibition halo expressed in millimeters (mm).

Although its antioxidant protective potential is evident due to the increased cellular enzymatic antioxidant activity, WJ-MSC-CM can also induce ROS production in dermal fibroblasts in response to oxidizing agent such as H_2_O_2_. Based on this observation, we hypothesized that WJ-MSC-CM possesses intrinsic antibacterial activity and that this activity may be enhanced following activation by H_2_O_2_. To demonstrate the antibacterial activity of WJ-MSC-CM, we performed agar well diffusion assay following the experimental scheme described in the Methods section. The tests were conducted using two bacterial strains: the Gram-positive *Staphylococcus aureus* and the Gram-negative *Pseudomonas aeruginosa*. The best results were obtained against *S. aureus*, as reported in **Figures 4A–C**. In all plates, the positive control produced a clear inhibition halo of 16 mm. In **Figure 4A**, Group 2 showed an inhibition zone comparable to the control, while Group 1 did not exhibit detectable antibacterial activity. In **Figure 4B**, neither Group 3 nor Group 4 produced an inhibition halo, indicating no observable inhibitory effect. In contrast, in **Figure 4C**, both Group 5 and Group 6 demonstrated clear antibacterial activity, with inhibition halos of 15 and 16 mm, respectively. Overall, the results indicate that Groups 2, 5, and 6 possess antibacterial activity, demonstrating a selective inhibitory activity against *S. aureus* of both WJ-MSC-CM alone and dermal fibroblasts treated with WJ-MSC-CM. Notably, when H_2_O_2_ were added to WJ-MSC-CM-treated fibroblasts (Group 6), *S. aureus* growth inhibition halo was similar to the vancomycin positive. No *P. aeruginosa* growth inhibition halo was detected (see **Supplementary Figure 1**).

Next, the antibacterial activity of active cell culture supernatants (Group 2, 5, and 6) against *S. aureus* was investigated in terms of minimum inhibitory concentration (MIC). As summarized in **Table 2**, Groups 2 and 5 exhibited antibacterial activity with a MIC at a 1:8 dilution, while Group 6 showed the strongest activity with a MIC at a 1:16 dilution. All together, these data demonstrate that WJ-MSC-CM contains antibacterial biofactors that inhibit the growth of *S. aureus* and that WJ-MSC-CM plays a key regulatory role in this activity when added to dermal fibroblast cultures. Furthermore, H_2_O_2_-stressed fibroblasts exert antibacterial effects in the presence of WJ-MSC-CM. Probably, when stressed fibroblasts are exposed to WJ-MSC-CM, they are stimulated to upregulate antimicrobial peptides, secrete antibacterial mediators such as ROS, and enhance innate immune responses.

**Table 2:** Antibacterial activity of test cell culture supernatants towards *S. aureus*.

	Strain	Group 1	Group 2	Group 3	Group 4	Group 5	Group 6	Positive control
MIC	*S. aureus*	–	1:8 dilution	–	–	1:8 dilution	1:16 dilution	1 µg/mL

### 3.5 Antibacterial effects of WJ-MSC-CM could be in part mediated from secretion of antimicrobial peptides

**Figure 5:**
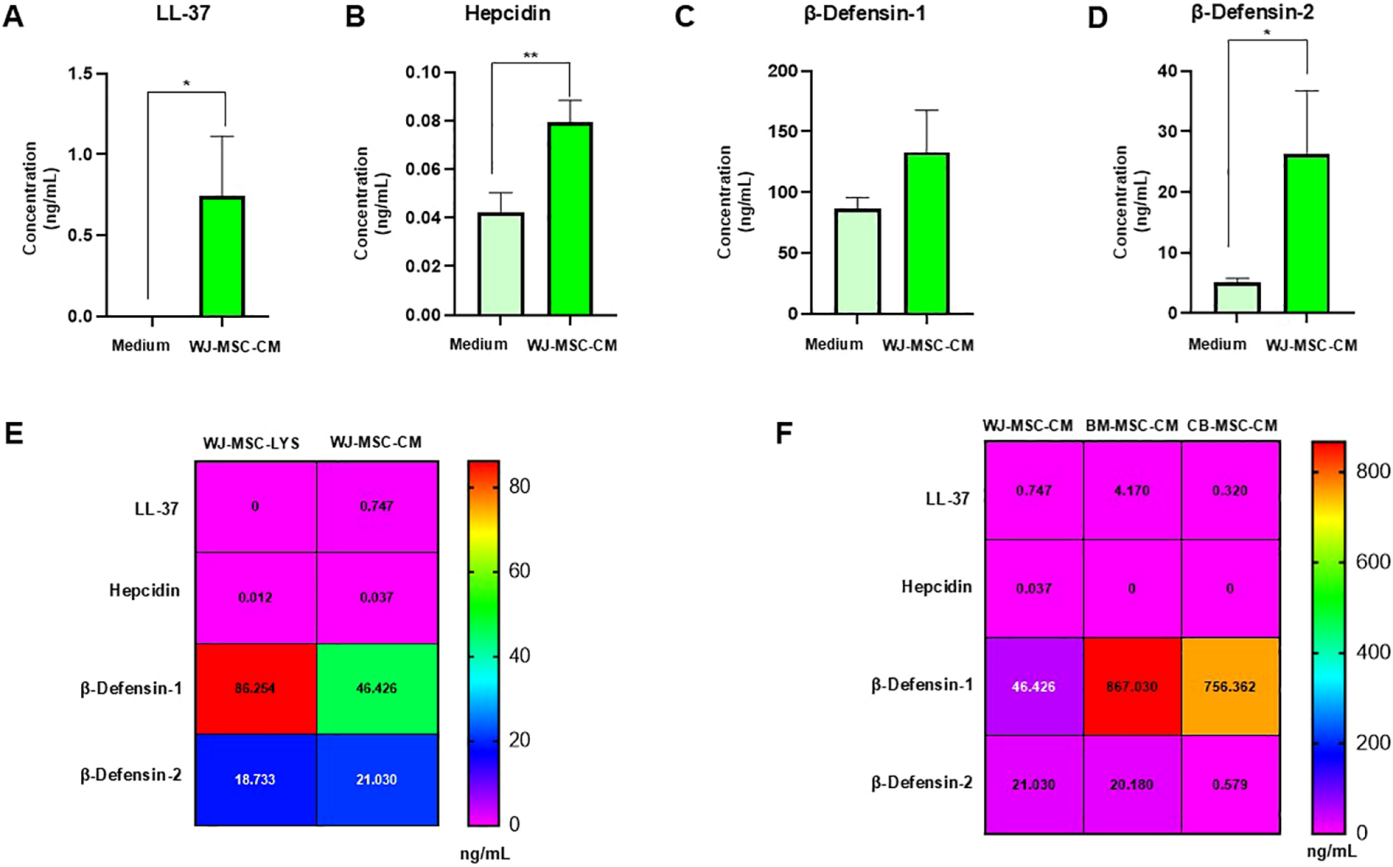
Quantitative analysis of antimicrobial peptides in WJ-MSC-CM. WJ-MSCs were cultured in basal medium, and conditioned medium (CM) was collected after 24 h. The concentrations of **(A)** LL-37, **(B)** hepcidin, **(C)** β-defensin-1, and **(D)** β-defensin-2 in the WJ-MSC-CM were quantified using ELISA kits. Data are presented as the mean of three independent samples. Statistical significance compared with the untreated control group is indicated as *p < 0.05. **(E)** Heatmap showing the mean basal levels (ng/mL) of three independent samples of LL-37, hepcidin, β-defensin-1, and β-defensin-2 detected in WJ-MSC lysates (WJ-MSC-LYS) and conditioned medium (WJ-MSC-CM) after subtraction of background signal from vehicle controls (lysis buffer for lysates; unconditioned basal medium for CM). **(F)** Heatmap showing the mean basal levels (ng/mL) of three independent samples of LL-37, hepcidin, β-defensin-1, and β-defensin-2 detected in WJ-MSC-CM, BM-MSC-CM, and CB-MSC-CM, after subtraction of background signal from unconditioned basal medium. **p < 0.01.

Since most preclinical studies have focused on bone marrow- and adipose tissue-derived MSC secretion of specific antimicrobial peptides (AMPs), such as cathelicidin LL-37, hepcidin, and β-defensins, we investigated whether also WJ-MSCs constitutively secrete these AMPs. To this end, the basal levels of LL-37, hepcidin, β-defensin-1, and β-defensin-2 in WJ-MSC-CM were quantified by ELISA. As shown in **Figure 5A**, LL-37 was undetectable in the control medium, while detectable basal levels were observed in WJ-MSC-CM (0.747 ± 0.36 ng/mL; **p < 0.02). The absence of LL-37 in the control medium indicates the adequate sensitivity and specificity of the ELISA kit, as it was able to clearly discriminate between the absence of the peptide in the medium alone and its presence in WJ-MSC-CM. Hepcidin levels were significantly increased (**p < 0.01) in WJ-MSC-CM (0.07 ± 0.008 ng/mL) compared with control medium (0.04 ± 0.008 ng/mL) (**Figure 5B**). For β-defensin-1, low levels detected in the basal medium (86.85 ± 8.97 ng/mL) may reflect background signal that the ELISA assay is unable to fully eliminate, whereas the higher concentrations measured in WJ-MSC-CM (133.28 ± 34.51 ng/mL) are indicative of specific peptide secretion (**Figure 5C**). Notably, β-defensin-2 levels were significantly higher in WJ-MSC-CM (26.34 ± 10.38 ng/mL) than in basal medium (5.05 ± 0.73 ng/mL), as reported in **Figure 5D**. Overall, these results indicate that, under basal conditions, WJ-MSC-CM contains low but detectable levels of antimicrobial peptides, which may contribute, at least in part, to its antibacterial activity.

To assess the regulation of AMP secretion by WJ-MSCs under basal conditions, the basal levels of LL-37, hepcidin, β-defensin-1, and β-defensin-2 in WJ-MSC lysates (WJ-MSC-LYS) and WJ-MSC-CM were compared. The heatmap in **Figure 5E** illustrates the mean basal levels (ng/mL) of AMPs detected in the WJ-MSC-LYS and WJ-MSC-CM, after subtraction of background signal from vehicle controls (lysis buffer for lysates and unconditioned basal medium for CM). Overall, a heterogeneous distribution of peptide levels is observed between the two sample types. LL-37 and hepcidin are present at very low levels in both WJ-MSC-LYS (0.000 ng/mL and 0.012 ng/mL, respectively) and WJ-MSC-CM (0.75 ng/mL and 0.037 ng/mL, respectively), with only minimal differences between the two compartments, suggesting limited basal production and/or secretion. In contrast, β-defensin-1 is markedly enriched in WJ-MSC-LYS (86.254 ng/mL) compared to WJ-MSC-CM (46.43 ng/mL), indicating that this peptide is predominantly retained intracellularly and may require specific stimuli to be efficiently released. Conversely, β-defensin-2 displays a more comparable distribution between lysate (18.733 ng/mL) and conditioned medium (21.03 ng/mL), suggesting a more efficient secretion and a potential role in mediating the extracellular antimicrobial activity of WJ-MSCs. Taken together, these findings indicate that WJ-MSCs differentially regulate AMP production and release under basal conditions, with β-defensins appearing to be the primary contributors to the antimicrobial activity among the selected AMP.3.6 Analysis of Antimicrobial Peptides in WJ-, BM-, and CB-MSC Conditioned Media.

A direct comparison of the basal levels of selected antimicrobial peptides released into the extracellular environment by MSCs derived from different tissue sources was performed. BM-MSCs were included as well-established and widely characterized MSC sources, commonly used as references, to determine whether the AMP profile observed in WJ-MSC-CM reflects a source-specific feature or a general property shared across MSC types. CB-MSCs were included as a fetal MSC model, similar to WJ-MSCs, to evaluate whether fetal-origin MSCs share common AMP secretion patterns distinct from adult-derived BM-MSCs.

The heatmap in **Figure 5F** illustrates the mean basal levels (ng/mL) of AMPs detected in the WJ-, BM-, and CB-MSC-CM, after subtraction of background signal from unconditioned basal medium. The highest level of LL-37 was observed in BM-MSC-CM (4.17 ± 3.43 ng/mL), followed by WJ-MSC-CM (0.75 ± 0.37 ng/mL) and CB-MSC-CM (0.32 ± 0.35 ng/mL). Hepcidin was largely undetectable, with only minimal levels observed in WJ-MSC-CM (0.037 ± 0.008 ng/mL). β-defensin-1 was the most abundantly detected peptide across all samples, with markedly elevated levels in BM-MSC-CM (867.03 ± 91.97 ng/mL) and CB-MSC-CM (756.36 ± 12.49 ng/mL), compared to substantially lower levels in WJ-MSC-CM (46.43 ± 34.51 ng/mL). β-defensin-2 exhibited similar levels in WJ-MSC-CM (21.03 ± 10.37 ng/mL) and BM-MSC-CM (20.18 ± 33.95 ng/mL), although BM-MSC-CM exhibited greater variability (as indicated by the large standard deviation). β-defensin-2 was nearly undetectable in CB-MSC-CM (0.58± ng/mL).

Collectively, these findings indicate that BM-MSC-CM displays the most pronounced AMP profile, primarily driven by high β-defensin-1 levels, whereas WJ-MSC-CM demonstrates a more balanced overall peptide expression pattern. CB-MSC-CM, while also exhibiting high β-defensin-1 levels similar to BM-MSC-CM, shows a more limited diversity of other AMPs, suggesting that fetal-origin MSCs may have distinct antimicrobial signatures depending on the source.

## 4 Discussion

WJ-MSC-CM has been evaluated for its effects in wound healing, tissue regeneration, and immunomodulation (18, 19). However, relatively little is known about their potential as antimicrobial agents. Therefore, the present study investigates the antibacterial properties of WJ-MSC-CM and their potential use in infection treatment.

Particularly, this work was initiated based on observations from the analysis of ROS production in dermal fibroblasts following WJ-MSC-CM treatment. Following infection or tissue damage, fibroblasts rapidly increase ROS production through NADPH oxidase and mitochondrial pathways. Beyond their antimicrobial function, ROS also act as signaling molecules that drive inflammation, promote cell proliferation, and initiate wound-healing processes (20). Recent studies have demonstrated that the secretome from AT-MSCs significantly reduces intracellular ROS levels in hepatic cells (21), while extracellular vesicles derived from umbilical cord MSCs (UC-MSCs) alleviate oxidative stress and enhance energy metabolism following ischemic injury (22). However, the effects of the WJ-MSC secretome on ROS production in dermal fibroblasts remain poorly characterized, and comprehensive data are currently lacking.

We observed that WJ-MSC-CM maintain low basal levels of ROS; however, following H_2_O_2_ exposure, WJ-MSC-CM–treated fibroblasts are able to produce high levels of ROS, which are not cytotoxic on the same cells, thus suggesting that WJ-MSC-CM could protects against oxidative stress-induced cell death in dermal fibroblasts (23), potentially via modulation of endogenous antioxidant defenses and redox-regulating pathways. Maintenance of redox homeostasis and prevention of excessive free radical production are ensured by cellular enzymatic antioxidants, including catalase (CAT), glutathione peroxidase (GPx), and superoxide dismutase (SOD). Specifically, these enzymes dismutate superoxide radical, breakdown hydrogen peroxides and hydroperoxides to harmless molecules (24). MSCs have been shown to enhance the antioxidant capacity of small bowel tissue following intestinal ischemia-reperfusion (I/R) injury by increasing the activity of CAT, GPx, and SOD (25). Moreover, antioxidant enzymes such as SOD, CAT, and GPx have been identified within BM-MSC-CM, supporting a paracrine mechanism underlying the antioxidant effects of MSCs (26).

Indeed, consistent with this redox-adaptive response, WJ-MSC-CM significantly enhanced the antioxidant activities of superoxide dismutase (SOD), catalase (CAT), and glutathione peroxidase (GPx). Firstly, these findings suggest that WJ-MSC-CM promote a controlled oxidative response while simultaneously strengthening endogenous antioxidant defenses, thereby maintaining redox homeostasis in dermal fibroblasts. Secondly, the controlled, non-cytotoxic increase in ROS, together with the enhancement of antioxidant defenses, may be indicative of a potential microbicidal effect. The concurrent increase in ROS levels alongside the upregulation of antioxidant defenses may initially appear paradoxical; however, it is more appropriately interpreted within the context of a dynamic redox-regulatory process rather than a strictly antagonistic effect. In this framework, moderate ROS production can act as a signaling event that triggers adaptive cellular responses, including the activation of endogenous antioxidant systems. Accordingly, WJ-MSC-CM may induce a controlled oxidative stimulus that promotes a compensatory antioxidant response, thereby contributing to the maintenance of redox homeostasis and cellular protection against oxidative stress. Thus, we examined the antimicrobial activity of WJ-MSC-CM against Gram-positive methicillin-resistant *Staphylococcus aureus* and Gram-negative *Pseudomonas aeruginosa*, using agar diffusion and MIC determination methods. WJ-MSC-CM showed no activity against *P. aeruginosa*, whereas it was active against MRSA, suggesting a selective antimicrobial effect, as already assessed by a previous study describing a preferential activity of AD-MSC secretome against Gram-positive bacteria (12). Therefore, this differential response may be attributed to structural differences in the bacterial cell envelope, as the outer membrane of Gram-negative bacteria acts as an additional permeability barrier that might limit the activity of soluble factors and antimicrobial peptides contained within WJ-MSC-CM. Interestingly, the antibacterial action of WJ-MSC-CM against MRSA was observed both with WJ-MSC-CM alone and with conditioned media from dermal fibroblasts treated with WJ-MSC-CM. This finding suggests that WJ-MSC-CM may induce secondary antimicrobial responses in recipient cells, potentially through the stimulation of antimicrobial peptide secretion or the modulation of innate immune pathways. H_2_O_2_-induced ROS production does not appear to be involved in the antimicrobial action, as conditioned medium from H_2_O_2_-stressed fibroblasts in control medium did not produce any inhibition zones. In contrast, conditioned medium from H_2_O_2_-stressed fibroblasts exposed to WJ-MSC-CM produced an inhibition halo comparable to that of vancomycin (positive control), suggesting that WJ-MSC-CM stimulates fibroblasts to increase their antibacterial activity in the presence of a pro-inflammatory signal such as H_2_O_2_. These observations imply that WJ-MSC-CM provides additional signals - possibly through secreted factors or paracrine modulation - that stimulate fibroblasts to upregulate antimicrobial peptides, secrete antibacterial mediators, or enhance innate immune responses.

We recently demonstrated that WJ-MSC-CM exhibits a superior regenerative profile compared to secretome of MSCs derived from other tissues, largely due to its higher content of growth factors and immunomodulatory cytokines (9). The high levels of IL-1β, IL-6, IL-17, and IFN-γ in WJ-MSC-CM could partially account for its antimicrobial properties observed in the present study, as these cytokines are known to activate innate immune responses, promote the recruitment and activation of neutrophils and macrophages, and stimulate the production of antimicrobial peptides (AMPs). Of particular interest are AMPs, as they represent key effector molecules of the innate immune response and may significantly contribute to the antimicrobial activity of the MSC secretome.

Accumulating evidence indicates that MSCs exert strong antimicrobial effects via both direct and indirect mechanisms (27). Indirectly, MSCs modulate the host immune response against pathogens; and directly, by the release of antimicrobial peptides (AMPs). AMPs, also known as host defense peptides, represent a broad class of endogenous molecules with both immunomodulatory and antimicrobial activities. These peptides act selectively against bacteria, yeasts, viruses, fungi, and cancer cells (28). Within the MSC secretome, specific AMPs - including cathelicidin LL-37, hepcidin, and β-defensins - have been identified and associated with bactericidal activity against *S. aureus* (27, 29). Although limited data are available on AMPs released by MSCs, recent *in vitro* studies have demonstrated that the antimicrobial effects of BM-MSCs are mediated by LL-37 (cathelicidin), β-defensins, and hepcidin (30, 31). Particularly, hepcidin - mainly known as a master regulator of iron homeostasis – has been considered as a key component of antimicrobial peptide arsenal in BM-MSCs (31). While previous studies have primarily focused on BM-MSCs and on intracellular or inducible AMP expression, evidence of their constitutive release within the secretome of perinatal MSCs remains limited. In this context, our findings extend current knowledge by demonstrating the presence of AMPs in WJ-MSC-CM. In line with these observations, our study demonstrates the simultaneous detection of LL-37, hepcidin, and β-defensin-2 in WJ-MSC-CM, whereas β-defensin-1 was predominantly retained intracellularly. The presence of both LL-37 and hepcidin suggests that WJ-MSC-CM may possess an intrinsic antimicrobial peptide repertoire. Notably, LL-37 and hepcidin exert complementary antimicrobial mechanisms: LL-37 directly disrupts microbial membranes and modulates immune cell recruitment, whereas hepcidin limits pathogen survival by regulating iron availability. β-defensin-2 may partially attenuate the epithelial barrier disruption induced by MRSA, indicating the protective effect against *S. aureus* infection (32). Beyond antimicrobial activity, these peptides are known to influence inflammation and tissue repair. LL-37 has been implicated in angiogenesis and wound healing, while hepcidin has been associated with immune regulation and inflammatory responses (33, 34). Recently, the potential of β-defensin-2–based hydrogels as a promising treatment for chronic wounds, such as diabetic foot ulcers (DFUs), has been demonstrated, owing to their antimicrobial, anti-inflammatory, and tissue-regenerative properties (35). Thus, their presence in WJ-MSC-CM may also contribute to the broader immunomodulatory and regenerative properties of the secretome of these cells. Of note, our data demonstrate that β-defensins are most abundant AMPs in WJ-MSCs. These epithelial-derived peptides are known to activate skin neutrophils, highlighting a potential role for this axis in both tissue homeostasis and the response to cutaneous infection (36). Given our focus on the development of WJ-MSC-CM-based hydrogels for chronic ulcers, these findings suggest that β-defensin-mediated antimicrobial and immunomodulatory activities may contribute to the therapeutic potential of WJ-MSC-CM in promoting wound healing and preventing infection. Additionally, WJ-MSC-CM exhibits a more heterogenous AMP pattern compared to CB-MSC-CM, confirming that among perinatal MSCs, WJ-MSCs may represent the superior source for immunomodulatory and antimicrobial peptides (9).This study is not without its limitations. First, the presence of LL-37, hepcidin, and β-defensin-2 was identified in the WJ-MSC-CM; however, their individual contribution to the observed antimicrobial activity was not directly assessed. Functional assays employing peptide neutralization would be required to elucidate the specific role of each antimicrobial peptide. Second, the composition of MSC secretome is known to be influenced by donor variability and culture conditions (37). Although standardized protocols were applied, potential variability in secretome content cannot be completely excluded and warrants further investigation. It would be desirable to analyze the gene expression of AMPs in WJ-MSCs and subsequently expand the proteomic analysis of the secretome, including additional factors such as lipocalin. In addition, the antimicrobial activity of the Wharton’s Jelly MSC secretome was evaluated against a limited panel of microorganisms, namely *S. aureus* and *P. aeruginosa*. Although these pathogens are clinically relevant and representative of Gram-positive and Gram-negative bacteria, respectively, the findings cannot be generalized to a broader spectrum of microorganisms. Future studies should extend the analysis to additional clinically relevant species, including Gram-negative Enterobacteriaceae and fungal pathogens, to better define the full antimicrobial potential of the secretome.

In conclusion, these results highlight the capacity of WJ-MSC-CM to modulate host cells and reinforce their potential as a novel strategy against *S. aureus*, one of the main pathogens responsible for persistent infections in surgical wounds and chronic ulcers, such as diabetic foot ulcers, colonizing mainly non-viable tissues and significantly delaying the healing process (38). The demonstrated activity against *S. aureus*, together with the detection of antimicrobial peptides such as LL-37, hepcidin, and β-defensins supports the relevance of WJ-MSC-CM as a multifunctional, cell-free therapeutic approach. These findings provide a foundation for further investigation into the translational application of WJ-MSC-CM in infection-associated and inflammatory conditions.

## Data Availability

The raw data supporting the conclusions of this article will be made available by the authors, without undue reservation.
