# Supplementary material for: The antimicrobial potential of the secretome from Wharton’s jelly mesenchymal stem cells in the context of regenerative medicine: an in vitro study
Source: Front Immunol. 2026 May 5;17:1818127. doi: 10.3389/fimmu.2026.1818127 (PMC13183550; doi:10.3389/fimmu.2026.1818127)
Supplement: Supplementary file 1 [file Presentation1.pptx]

## Slide 1
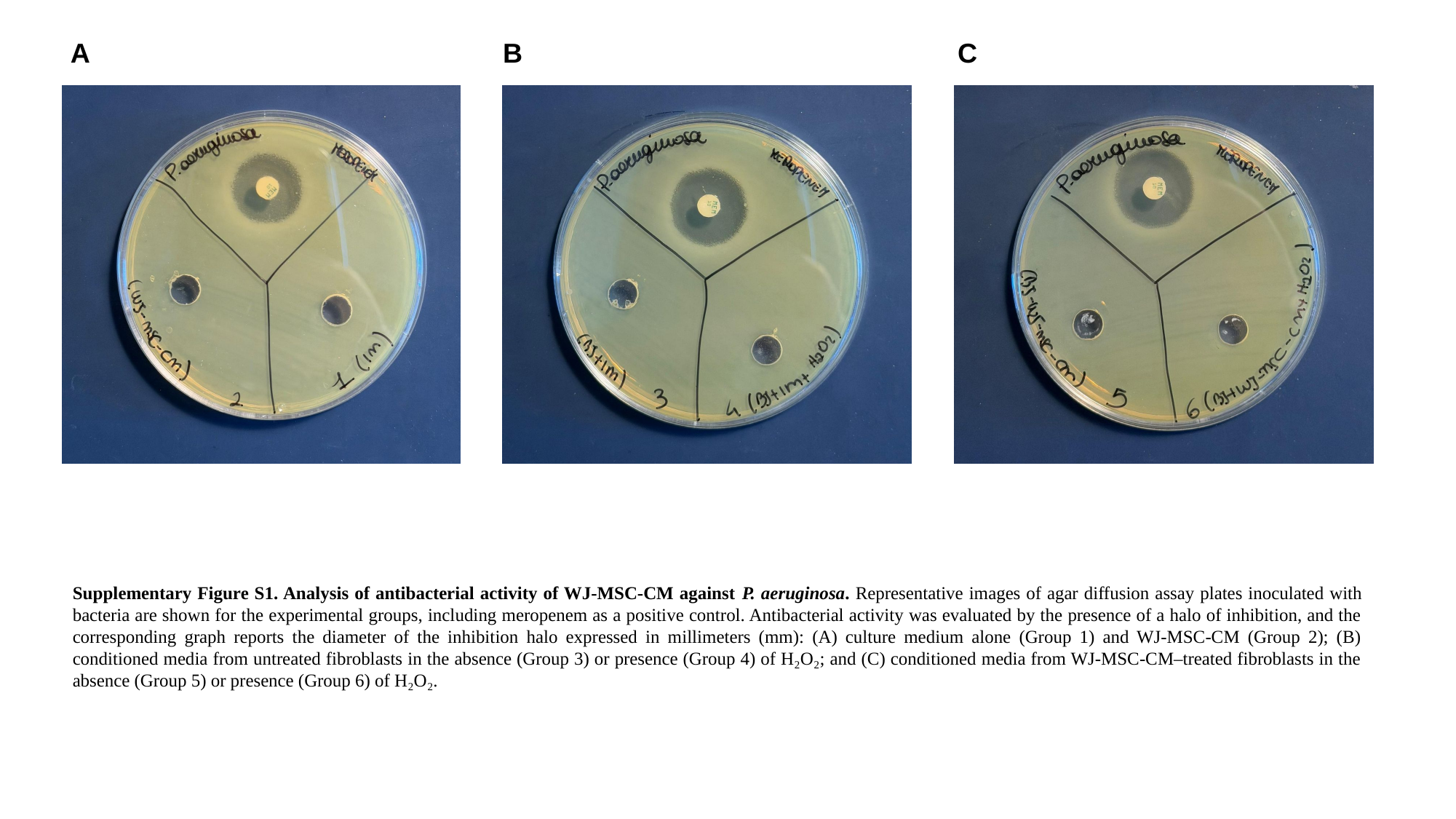

B
C
A
Supplementary Figure S1. Analysis of antibacterial activity of WJ-MSC-CM against P. aeruginosa. Representative images of agar diffusion assay plates inoculated with bacteria are shown for the experimental groups, including meropenem as a positive control. Antibacterial activity was evaluated by the presence of a halo of inhibition, and the corresponding graph reports the diameter of the inhibition halo expressed in millimeters (mm): (A) culture medium alone (Group 1) and WJ-MSC-CM (Group 2); (B) conditioned media from untreated fibroblasts in the absence (Group 3) or presence (Group 4) of H₂O₂; and (C) conditioned media from WJ-MSC-CM–treated fibroblasts in the absence (Group 5) or presence (Group 6) of H₂O₂.
